# The impact of terminating cost-sharing reductions payments on health insurance plan choices

**DOI:** 10.3389/fpubh.2024.1370563

**Published:** 2024-05-10

**Authors:** Fanyu Liu

**Affiliations:** Department of Economics, Tulane University, New Orleans, LA, United States

**Keywords:** health reform, advanced premium tax credits, cost-sharing reductions, health insurance, affordable care act

## Abstract

The Trump administration terminated cost-sharing reductions (CSRs) payments to health insurers in 2017, while still required insurers to provide CSRs to eligible enrollees in the Marketplace. Marketplace administration data reveals that, in response to this termination, insurers raised premiums to compensate for their loss. Consequently, premium increases led to more advanced premium tax credits for enrollees in the Marketplace. To investigate the impact of CSRs payment termination on low-income consumer insurance plan choices, I leverage variations in state price regulations and employed a difference-in-differences design. In a robustness analysis, I utilized differences in county income distributions to examine the effects of the termination on insurance choices. The results indicate that after the termination, more low-income enrollees opted for cheaper bronze plans, and fewer chose silver plans. These results suggest that alterations in subsidy channels may inadvertently encourage low-income individuals to purchase less expensive health insurance plans, highlighting an unintended consequence of the termination of cost-sharing subsidies.

## 1 Introduction

The 2010 Affordable Care Act (ACA) was enacted to expand health insurance coverage and reduce financial barriers to obtaining insurance for low-income individuals. One of the key provisions of the ACA was the establishment of the health insurance exchange (Marketplace) in 2014. The Marketplace allows people with family incomes between 100% and 400% of the federal poverty line (FPL) to purchase private health insurance with the assistance of two types of government subsidies: advanced premium tax credits (APTCs) and cost-sharing reductions (CSRs). APTCs are designed to reduce the monthly premium payments for insurance plans purchased through the Marketplace, while CSRs aim to lower out-of-pocket costs, such as copayments, for people with family incomes below 250% of the FPL and enrolled in the silver plan. Literature demonstrates that consumers are sensitive to the CSRs. Researchers found people with family incomes under 250% of the FPL were more likely to purchase the silver plan (1), and showed that the coverage rates just below 200% of the FPL are significantly higher than coverage rates above 200% of the FPL (2).

However, on October 12, 2017, the Trump Administration terminated the payment of CSRs to health insurers, while insurers were still required to provide CSRs to eligible enrollees (3). This change may result in increased costs for insurance companies. In response, insurers raised premiums to compensate for the additional expenses. Health policy experts and insurance company representatives have pointed out that the premium increases in the Marketplace in 2018 were largely attributed to the termination of CSRs payments to insurers (4, 5). An unintended consequence of terminating CSRs is that consumers might receive more APTCs based on the mechanics of APTCs, thus being encouraged to purchase Bronze, Gold or Platinum plans.^1^ This paper attempts to quantitatively analyze the impact of CSRs termination on consumers' choices across different metal plans.

According to estimates by the Kaiser Family Foundation, in response to the termination of CSRs, most insurers raised the premiums of the silver plan to compensate for the loss in payments (6). In 2018, on average, the lowest silver plan's premium for a 27-year-old increased by 32%, while the premiums for the lowest bronze and gold plan only increased by 17% and 18%, respectively, across all states. As a result, consumers might receive more APTCs since the APTCs are determined by the difference between the premium of the second-lowest silver plan and the premium cap (the maximum percentage of income one must pay for the second-lowest silver plan available in their area). In 2018, the premium cap for people with family incomes between 150% to 200% of the FPL was 4.03% to 6.34% (7), and the range in 2017 was 4.08% to 6.43% (8). Therefore, given the premium caps are roughly the same in 2017 and 2018, an increase in the premium of the second-lowest silver plan could lead to consumers receiving more APTCs. Then, the additional APTCs could be applied to any plan available in the Marketplace, including bronze, gold, and platinum plans, thereby reducing the after-APTCs price of these plans for consumers.

Literature shows that after the termination of CSRs payments to insurers, more counties had zero-dollar premium (after deducting the APTCs) plans, which prompted low-income people to enroll in such plans (9). For example, in 2018, a 40-year-old with a household income of $25,000 could purchase a zero-premium plan in 1,679 counties, whereas in 2017, such a plan was available in only about 220 counties (10).

**Figure 1:**
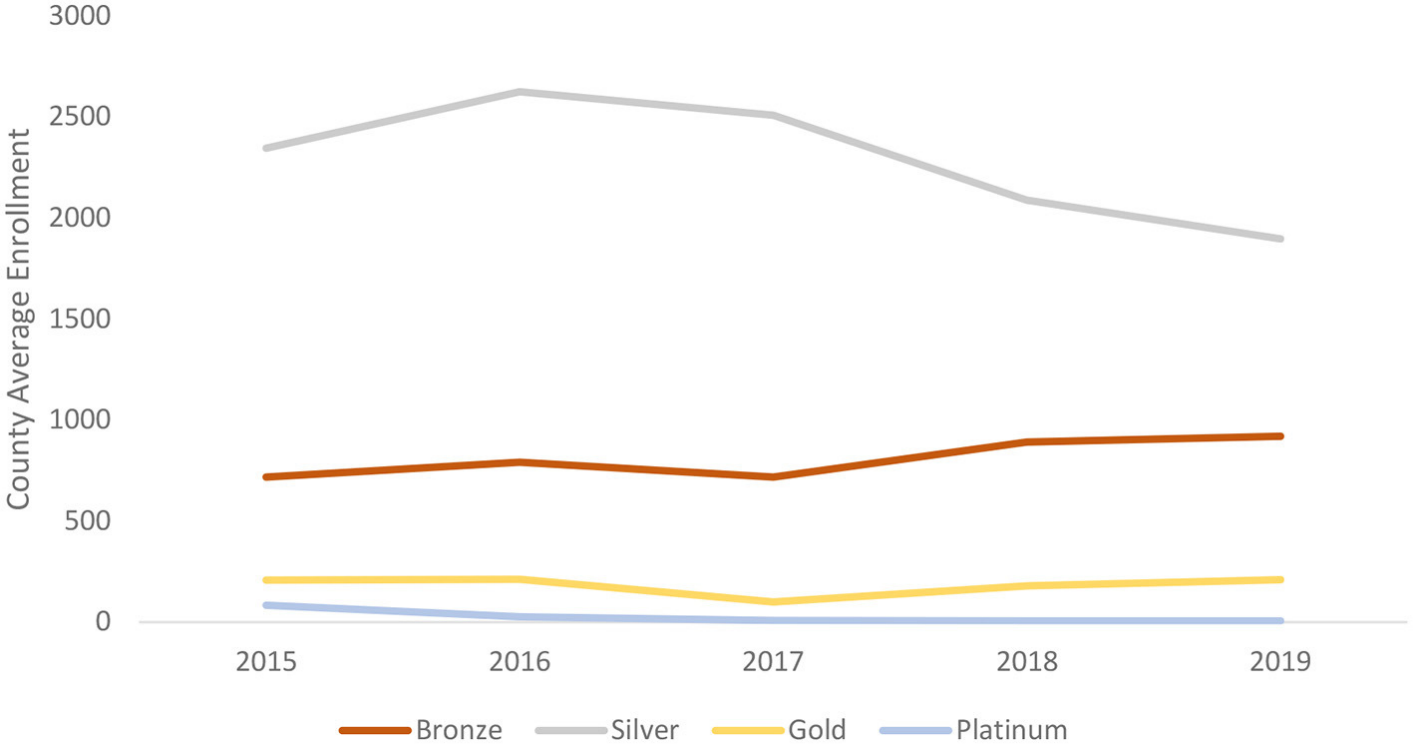
The county average enrollments of each plan since 2015 to 2019. Sources: Authors' analysis of data for 2015–19 from the open enrollment period public use files.

Figure 1 shows the average number of enrollees in each plan in each county from 2015 to 2019. After 2017, the average number of enrollees in the silver plan declined, while the number of enrollees in the bronze plan rose significantly. In addition, there was a small increase in the enrollments in the gold and platinum plans.

In this study, I use variations in state premium regulations and employ a difference-in-differences (DiD) design to identify the impact of the termination of CSRs payments on consumers' insurance plan choices. In the states that only allow insurers to increase the premium of the silver plan inside the Marketplace, the decline in enrollment in the silver plan and the increase in enrollment in the bronze plan were more significant than those allowing insurers to raise the premiums of all plans, while there were no significant changes in enrollments in the gold and platinum plans. On average, the enrollments in the bronze plan increased by 450 (increased 61% compared with the average enrollment before the termination), while the number of enrollees in the silver plan decreased by 333 (decreased 13% compared with the average enrollment before the termination) in each county. Additional findings indicate that the decrease in silver plan enrollment was more pronounced in counties where a larger percentage of the population has family incomes exceeding 200% of the FPL. This is because this group is less likely to benefit from CSRs, leading them to opt out of the silver plan. When the proportion of enrollees with family incomes above 200% of the FPL increases by 1%, the share of enrollees in the silver plan decreases by 0.34%, and the share of enrollees in the bronze plan increases by 0.32%.

This study contributes to the economics literature on the impact of healthcare system subsidies on consumer behaviors by examining the impacts of the termination of CSRs and sheds light on the effects on consumers' purchasing behavior when subsidy channels for health insurance are altered. The findings highlight that altering subsidy channels may have unintended consequences. Despite receiving more APTCs after the termination of CSRs payments, many enrollees switched to cheaper bronze plans instead of gold or platinum plans. While this reduced the one-time expense of purchasing insurance, it also resulted in low-income people receiving low-quality health insurance plans. This study complements findings from similar studies that low-income people's willingness to pay for health insurance is always less than the reimbursement they expect to receive from insurance companies, and that low-income people are more likely to drop out of the health insurance market when health insurance subsidies are reduced (11). A potential reason for this result could be that low-income groups face lower budget constraints and therefore have to shift resources from the purchase of high-quality health insurance to the consumption of other goods. Research in this area could provide valuable insights for policymakers and help inform future policy decisions related to health insurance subsidies.

## 2 Policy background

**Figure 2:**
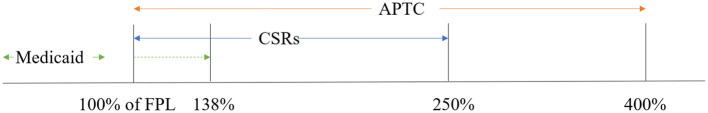
ACA subsidies schedule.

Figure 2 depicts the subsidies schedule of ACA. The ACA consists of two main parts, Medicaid expansion and the Marketplace. The solid green line represents the family incomes range for which Medicaid is available in non-expansion states and the dotted line represents the family incomes range in expansion states. In expansion states, people with family incomes below 138% of the FPL are eligible for Medicaid, while in non-expansion states, the incomes thresholds for eligibility vary but are below 100% of the FPL. Studies show that the ACA significantly improves health insurance coverage (12, 13), health conditions (14), and household finances for low- and middle-income populations (15–17).

People who do not have employer-sponsored health insurance and are not eligible for Medicaid or Medicare may be eligible for ACA Marketplace subsidies when they meet certain family incomes limits. Specifically, people with family incomes between 100% and 400% of the PFL are eligible for APTCs (in Medicaid expansion states, the lower bound is 138% of the FPL). APTCs depends on the difference between the second-lowest silver plan and the insurance premium cap. The premium cap rises as a family's income rises. Because the ACA does not allow for different insurance premium to be set for different incomes in a given region, the actual APTCs received by a family decrease continuously as income increases.

Second, people with family incomes between 100% and 250% of the FPL and enroll the silver plan are eligible for CSRs. Table 1 depicts the subsidy rates of CSRs for different income group. Actuarial value means the percentage of the bill that is covered by the insurance company. For enrollees without CSRs, the actuarial value is 70%, which implies that for every $100 medical bill, the enrollees pay $30 and the insurers cover the rest $70. For enrollees with CSRs, the actuarial value of the silver plan increases as the family income falls. For people with family income between 100% and 150% of the FPL, the subsidy from CSRs is 24%, for people with family income between 150% and 200% of the FPL, the subsidy is 17%, and for people with family income between 150% and 200% of the FPL, the subsidy is only 3%.

**Table 1:** CSRs subsidy rates in 2018.

**Income (% of the FPL)**	**Actuarial value of a silver plan**
100%–150%	94%
150%–200%	87%
200%–250%	73%
Over 250%	70% (no CSRs)

Table reports the actuarial value of a silver plan in 2018. Source: www.federalregister.gov/documents/2020/05/14/2020-10045/.

## 3 Data sources

This study combines data from the Marketplace Open Enrollment Period Public Use Files (PUFs)^2^ and the Small Area Income and Poverty Estimates Program (SAIPE)^3^ through the U.S. Census. The PUFs summarize data in the Multidimensional Insurance Data Analytics System (MIDAS) from 2015 to 2019, including how many people enrolled in each metal plan at the county level and demographic characteristics (age interval, race, and income interval) within a county's Marketplace. In sum, I have 13,458 observations at the county-year county level. As shown in Table 2, the average number of enrollees in the silver plan in a county decreased from 2,491 to 1,847, while the average number of enrollees in bronze plans in a county increased from 741 to 959 after the termination. It is worth noting that the total number of enrollees in the ACA Marketplace also declined after 2017, which might mean that people are exiting the Marketplace.

**Table 2:** Summary statistics.

**Variable**	**All periods**	**Before termination**	**After termination**
Total enrollment	3,321.82 (13,641.63)	3,477.96 (13,696.6)	3,165.66 (13,585.52)
Number of enrollees (Silver)	2,256.65 (9,987.60)	2,491.63 (10,242.77)	1,847.36 (9,516.94)
Number of enrollees (Bronze)	855.46 (3330.17)	741.53 (2823.21)	959.08 (3,768.92)
Number of enrollees (Gold)	187.45 (641.96)	171.06 (669.82)	203.74 (595.76)
Number of enrollees (Platinum)	22.27 (192.93)	38.71 (266.21)	5.54 (54.36)
Average age of exchange enrollees	41.5 (5.24)	41.26 (5.26)	41.74 (5.42)
Ratio of white people to all enrollees in each exchange	0.60 (0.18)	0.56 (0.18)	0.65 (0.17)
Ratio of people with family incomes above 200% FPL to all enrollees in exchange	0.46 (0.15)	0.57 (0.14)	0.50 (0.15)
Ratio of people with family incomes above 200% FPL to county population	0.80 (0.01)	0.80 (0.01)	0.80 (0.01)
County population	753,638 (1,955,806)	661,978 (198,792,0)	608,183 (1,822,740)
#Sample	13,458	7,922	5,536

The above table shows summary statistics among 2015 to 2019. The total observation is 13,458. All data are averaged at the county level.

The PUFs separate enrollees into eight races (American Indian, Asian, African American, Latino, Native Hawaiian, Multiracial, White, Unknown Race). I used these data to calculate the proportion of white enrollees in the total enrollment as an estimate of the majority proportion. This dataset also reports age in intervals. I take the midpoint of each interval and then multiply it by the number of enrollees in each interval. After adding these intervals together, I divide them by the total number of enrollees to calculate the average age in each county. The SAIPE contains county-level population data and income distribution. The data used in this paper includes 36 states that report statistics of their Marketplaces to the Centers for Medicare and Medicaid Services (CMS), which operates the PUFs. The states that run their own Marketplaces and do not report data are excluded.

## 4 Empirical design

### 4.1 State premium regulations

In this study, I first leverage the variation in state price regulation policies to identify the causal impact of CSRs termination on consumers' plan choices. In 2018, there are four main kinds of price regulation policies (18).

1. State laws do not allow insurers to adjust premiums in response to the termination of CSRs payments (3 states).
2. States allow insurers to increase premiums for all plans inside and outside the marketplace (6 states).
3. States allow insurers to increase silver premiums inside and outside the marketplace (21 states).
4. States allow insurers to increase silver premiums inside the marketplace because only the silver plans inside the ACA marketplace are affected by the CSRs (21 states).

Only three states did not adjust rates at all in response to the termination of the CSRs payments. Approximately half of the states allowed increased premiums for all plans inside and outside the Marketplace (broad loading) or increased premiums of silver plans inside and outside the Marketplace (silver loading). The other half only allowed increasing silver plans' premiums inside the Marketplace because only the silver plans inside the Marketplace are affected by CSRs.

For the states that adopted policies one, two, and three, the premium increases on consumers outside the Marketplace could offset some loss of the CSRs inside the Marketplace. Thus, the premium increase of silver plans should be lower than that of the states that only allow insurers to increase silver plans' premiums inside the Marketplace. PUFs data show that the average percentage premium increase of the second-lowest silver plan for a 27-year-old individual in treated states was 47%, while the percentage increase in control states was 31% in 2018.^4^ Based on the mechanics of APTCs, consumers in states that adopted policy four receive relatively more extra APTCs; thereby, changes in enrollments in each plan in these states should be larger. The variations in states adopted different policies allow me to use a standard DiD design to identify the causal impact on enrollment in each metal plan.


(1) $$\begin{array}{l} y_{it}=\alpha +\beta Post_{t}\;\cdot \;Treated_{i}+\gamma X_{it}+\mu_{i}+\theta_{t}+\varepsilon_{it} \end{array}$$

In Equation 1, the treated groups are states that only allow insurers to increase premiums of the silver plan inside the Marketplace (policy four). The control groups are states that allow insurers to increase silver premiums both in and out of the Marketplace or premiums for all plans (policies one, two, and three).^5^ In summary, there were 12 states in the treated group and 23 states in the control group. The outcome variable *y*_*it*_ is the number of enrollees in county and year of each plan (bronze, silver, or the sum of the gold and platinum plans). Because the termination started in 2017, I set post periods equal 2018 and 2019. Besides the DiD intersection term, four independent variables are controlled, including the average age in the county's Marketplace, the percentage of white people in the Marketplace, the proportion of people with family incomes above 200% of the FPL in each Marketplace, and the county's population. I control county and year fixed effects to absorb characteristics that do not vary across counties over time and do not vary geographically within each year. The coefficient of interest is β, which measures whether insurers who were only allowed to increase the silver premium inside the Marketplace encourage more enrollees to choose the bronze plan and fewer enrollees to choose the silver plan.

It is worth noting that since people could only choose one of the four types of health insurance in the Marketplace. When more people chose the bronze plan, it inevitably led to fewer enrollees in the other plans (assuming no people switch out or in the Marketplace). That is, there is perhaps correlation in the error terms of the regression. Therefore, I show the results of regressions using the Seemingly Unrelated Regressions in the Appendix to increase the robustness of the empirical results.

### 4.2 Proportion of low-income population by state

Next, using a modified form of the standard DiD design, I examine the impact on metal plan enrollment by employing two indicators to measure treatment intensity. First, I calculate the ratio of the number of enrollees in each county with family incomes above 200% of the FPL to the total number of Marketplace enrollees in that county in 2017. According to PUFs, in 2017, 75.6% of enrollees with family incomes below 200% of the FPL received CSRs. For counties with a higher proportion of enrollees with incomes above 200% of the FPL, changes in the number of enrollments in each plan might be more pronounced than counties with a smaller proportion of participants with incomes above 200% of the FPL. Because enrollees with incomes below 200% of the FPL and choose the silver plan could receive CSRs, they have less incentive to switch to the other three plans. As shown in Table 1, although people with family incomes between 200% and 250% of the FPL could receive CSRs, according to the subsidy schedule of CSRs, the actuarial value of a silver plan for such income group is 73%, which is just slightly higher than enrollees who do not receive CSRs (the actuarial value is 70%). Therefore, in the modified DiD model, the ratio of the number of enrollees with family incomes above 200% of the FPL to the total number of enrollees in 2017 is used as the measurement of treated intensity (the actuarial value for the 150% to 200% group is 87%).

Another indicator is the ratio of people with family incomes above 200% of the FPL to the whole county population. Compared with the ratio of enrollees, the ratio of the county population is determined by the county's characteristics and is not impacted by the Marketplace's policy. Thus, the ratio of the county population is exogenous to the Marketplace.


(2) $$\begin{array}{l} y_{it}=\alpha +\beta Post_{t}\;\cdot \;Proportion_{i}+\gamma X_{it}+\mu_{i}+\theta_{t}+\mu_{it} \end{array}$$

In the modified DiD model (Equation 2), outcome variables are the ratio of the number of enrollees in the silver, bronze, or the sum of gold and platinum plans to the total number of enrollees in the Marketplace, and the coefficient β measures the treatment intensity, which equals the proportion of enrollees with family incomes above 200% of the FPL or the ratio of people with family incomes above 200% of the FPL to the whole county population in 2017. The other variables are the same as in the standard DiD model.

One potential threat to measurement is that if one county has more enrollees with family incomes below 200%, the increase in premium for the silver plan is also likely to be higher, as insurers need to compensate more for losses from CSRs. Thus, enrollees may receive more APTCs, encouraging them to switch to bronze, gold, or platinum plans. To eliminate this threat, I regress the premium increase on the proportion of enrollees with income above 200% of the FPL.


(3) $$\begin{array}{l} y_{i}=\alpha +\delta Proportion_{i}+\gamma X_{it}+\mu_{i} \end{array}$$

In Equation 3, *y*_*i*_ is the difference between the average surcharge of the second-lowest silver plan or the surcharge of the lowest bronze plan. *Proportion*_*i*_ is the proportion of enrollees with family incomes above 200% FPL. *X*_*it*_ is a vector of covariables, which includes average age, the proportion of white people, and the county population. If the estimation of coefficient δ is not significant or not away from zero, it may imply the premium surcharges is not determined by the proportion of enrollees with family incomes above 200% FPL in each county.

### 4.3 Dynamic effects over time and parallel trend assumption tests

My identification assumption requires that if the CSRs to insurers had not terminated, the change in the enrollment in each metal plan would have the same rate. Thus, I use an event study style regression to test the parallel trend assumption (Equation 4). The base year is 1 year before the treatment is implemented. If estimates of the before-treated periods (i.e., β_*t*_) are not significant and away from zero, we cannot reject the premise of a common trend between the treated and control groups. The other variables are the same in Equation 1.


(4) $$\begin{array}{l} y_{it}=\alpha +\overset{2019}{\underset{t=2015}{\sum }}\beta_{t}Post_{t}\;\cdot \;Treated_{i}+\gamma X_{it}+\mu_{i}+\theta_{t}+\varepsilon_{it} \end{array}$$

## 5 Empirical results

### 5.1 Impacts of price regulations on enrollments

Table 3 shows impacts of different price regulation policies on number of enrollees in each plan. Compared with states that allow insurers to increase premiums inside and outside the Marketplace, the states that allow increases the premium of the silver plan only inside the Marketplace have more pronounced changes in enrollments in each plan. The enrollment in the bronze plan increased by 450, and the number of enrollees in the silver plan decreased by 331 on average. In the control states, insurers could increase the silver premiums in and out of the Marketplace and even increase the premiums of all plans. Thus, the premium increases of silver plans in the Marketplace are less than those in the treated states. Therefore, enrollees receive more APTCs in the treated states, which encourages them to leave the silver plan and choose the other three metal plans. However, the change in the gold and platinum plans is positive but not significantly different than zero. One possible explanation is people in the Marketplace generally are low- and mid-income people. Compared with choosing an expensive plan with a broader coverage range, they are more price sensitive and prefer a cheap plan, in other words, the bronze plan.^6^

**Table 3:** The impacts of different price regulation policies on number of enrollees in each plan.

	**(1)**	**(2)**	**(3)**
**Enrollment**	**Silver**	**Bronze**	**Gold and platinum**
Post^*^Treated	−333.11^*^	450.41^*^	10.08
	(183.09)	(246.06)	(63.93)
200% FPL Income Ratio	337.20^***^	−410.22^*^	141.48^**^
	(103.02)	(236.24)	(59.68)
Population	−1,511.61	2,524.85	−461.15
	(907.69)	(1,758.31)	(475.33)
Age	−33.29^***^	−0.02	−4.73
	(10.15)	(3.41)	(3.44)
White Prop	−264.25	−644.03^**^	−46.71
	(229.75)	(250.67)	(84.74)
Observations	12,489	12,489	12,489
Mean	2491.63	741.53	171.06

Authors' analysis of data for 2015–19 from the Open Enrollment Period Public Use Files. Table shows the did estimates of the impacts of different price regulation policies on number of enrollees in each plan from 2015 to 2019. Standard errors are reported in parentheses and clustered at the state level. ^***^p < 0.01, ^**^p < 0.05, ^*^p < 0.1.

### 5.2 Effects of income level on enrollment ratios

Next, I show the regression results of Equation 2 using the proportion of people with family incomes above 200% of the FPL in the Marketplace as the measure of treated intensity. When the proportion of enrollees with family incomes above 200% of the FPL increases by 1%, the share of enrollees in the silver plan decreased by 0.34% and the share of enrollees in the bronze plan increased by 0.32%. The share of enrollees in the gold and platinum plans increased by 0.02% while it is not significant than zero. Enrollees with family incomes below 200% of the FPL can access CSRs, so they are more likely to stay in the silver plan rather than choose the other three metal plans. Thus, when the proportion of enrollees with family incomes above 200% of the FPL in a county increase, the share of the silver plan decreases, and the share of the bronze plan increases. Since low- and mid- income people are more sensitive to the price of insurance, they prefer to switch down to the bronze plan rather than choosing the gold and platinum plans. Using the ratio of people with family incomes above 200% of the FPL as the treatment intensity produces similar results as shown in Table 4, Panel A.

**Table 4:** The impacts on enrollments proportion in each plan.

**Panel A: The effect of proportion of low-income people in each Marketplace on changes of enrollments**.			
	(1)	(2)	(3)
Enrollment Ratio	Silver	Bronze	Gold and Platinum
Post^*^ Proportion	−0.34^***^	0.32^***^	0.02
	(0.10)	(0.08)	(0.08)
Observations	12,871	12,871	12,871
R-squared	0.75	0.64	0.49
**Panel B: The effect of proportion of low-income people in each county on changes of enrollments**.			
	(1)	(2)	(3)
Enrollment Ratio	Silver	Bronze	Gold and Platinum
Post^*^ Proportion	−2.42^***^	1.60^**^	0.82
	(0.87)	(0.59)	(0.81)
Observations	12,871	12,871	12,871
R-squared	0.75	0.63	0.49

Table shows the fuzzy did estimates of the effect of income proportion in each county or Marketplace on changes of enrollments for 2015 to 2019. Estimations of control variable are omitted in the Table. Standard errors are reported in parentheses and clustered at the state level. ^***^p < 0.01, ^**^p < 0.05, ^*^p < 0.1.

As discussed above, if one county has more enrollees with family incomes below 200%, the premium increase of the silver plan may also be larger because insurers need to compensate more. Thus, I use the Equation 3 to examine the impact of the proportion of enrollees with family incomes above 200% of the FPL on the difference between the average premium increases of the second-lowest silver plan or the premium increases of the lowest bronze plan. The coefficient is −161.13 (95.02), which indicates the proportion had no significant impact on the difference between the silver premium increases and the bronze premium increases. Thus, although a larger proportion of enrollees with family incomes below 200% of the FPL may generate more cost to insurers, the extra-costs were not reflected in the difference between premium increases of the silver plan and the bronze plan (i.e., the differences between increases were not significantly different among counties). Therefore, enrollees in counties with a larger proportion of enrollees with family incomes below 200% of the FPL did not have more incentive to switch to the other plans.

**Figure 3:**
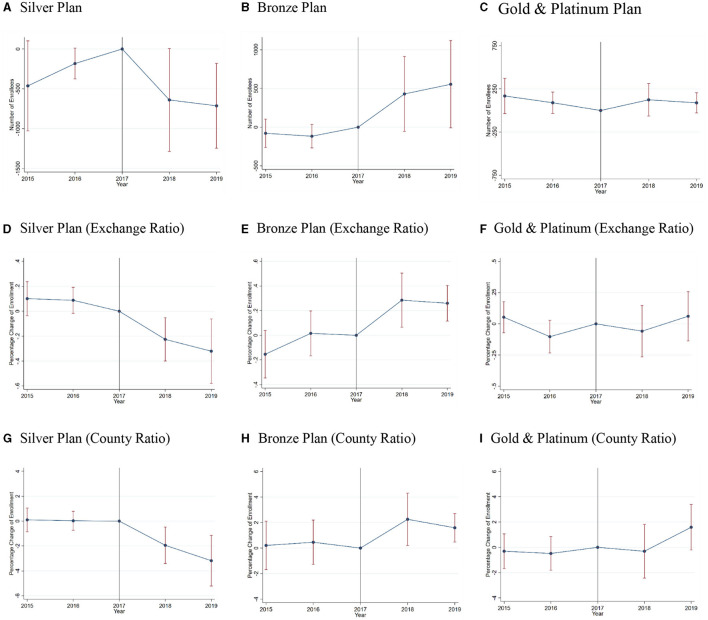
Event studies for the parallel trend assumption. Authors' analysis of data for 2015–19 from the Open Enrollment Period Public Use Files. This Exhibit shows the estimation results of the parallel trend assumption for the enrollment in each metal plan. The base year is 2017, 1 year before the termination of CSRs. **(A–I)** show the number/ratio of enrollees change after the CSRs termination. Exchange Ratio represents using the proportion of people with family incomes above 200% PFL to the enrollment in exchange as the measure of treatment intensity. County Ratio represents using the proportion of people with family incomes above 200% PFL to the county population as the measure of treatment intensity.

One fundamental assumption of the DiD model is the parallel trends assumption. The parallel trends assumption ensures that the treated and control groups have a common trend. Following figures show the test results of the parallel trend assumption by Equation 4. The base year is 2017, 1 year before the termination of CSRs to insurers. As shown in Figure 3, the *p*-values of the *t-*tests for all scenarios in each period before treatment were not significant at the 90% level. Thus, the results cannot reject the premise of a common trend between groups.

## 6 Conclusion

This study analyzes the impact on low-income consumers' behavior if the government alters the subsidy channels of purchasing health insurance. More specifically, I evaluate the impact of the cut in CSRs for insurers on Marketplace plan enrollment. The empirical results find that among the 36 states that report Marketplace enrollment statistics to the CMS, states that only allowed higher premiums for silver plans to make up for insurers' loss had an average of 450 more people per county choosing the bronze plan, while an average of 333 fewer people chose the silver plan than in the other states. The empirical estimations also show that when the proportion of enrollees with family incomes above 200% of the FPL increases by 1%, the share of enrollees in the silver plan decreases by 0.34%, and the share of enrollees in the bronze plan increases by 0.32%.

After 2018, the government stopped paying CSRs to insurers but still required insurers to provide CSRs to consumers in the Marketplace. For consumers, the CSRs they received did not change. However, the insurers increased premiums (especially the silver premium in the Marketplace) to compensate for the CSRs payment termination, which generated more APTCs for consumers, encouraging them to enroll in the bronze plan rather than stay in the silver plan. Since enrollees in the Marketplace are low- and mid-income people, they are likely to be more sensitive to the price of insurance and might not be able to maximize their utility. Thus, they might prefer to choose a cheaper plan, rather than the gold and platinum plans even if these plans have lower copayment rate and broader cover range (19, 20).

This study provides a reference for policymakers and researchers aiming to encourage low-income people to purchase appropriate health insurance. After the government terminated the CSRs payments, insurers increased premiums. Thus, the government just transferred the subsidy from CSRs to APTCs and the total government expenditure on the Marketplace might also increase. The Congressional Budget Office reports that between 2017 and 2026, the policy is projected to increase spending on APTCs by $365 billion, while spending on CSRs is expected to decrease by only $118 billion (21). However, this adjustment induced many enrollees to choose a lower-level plan (from silver to bronze), which is an unintended consequence of the government's policy action. This study also helps insurers price the insurances.

Unfortunately, this study cannot track the consumption behavior of specific individuals. If subsequent studies access individual-level panel data, the results will provide more accurate information. For example, we can observe whether individuals switch from the silver plan to another plan or not. It is also possible to examine the heterogeneity effect of ACA Marketplace enrollees across income ranges. This paper also lacks discussion of health insurance markets outside the Marketplace. From 2017 to 2018, the average enrollment in the silver plan decreased by 644 in county, while the average enrollment in the sum of the platinum, gold, and bronze plans increased by 217, which implies people left the Marketplace after the termination of CSRs payments. One extension of this study is exploring impacts on uninsured people and people who purchase health insurance outside the Marketplace.

Another meaningful extension of this study is whether different policies and market structures have different impacts on government spending. Literature shows that in counties with monopoly insurers, the silver plan premium increase should be larger (22); meanwhile, in states that allow insurers to only increase premiums inside the Marketplace, the premium increases of the silver plan should be larger as well, which implies higher expenses on APTCs. After evaluating the impact on government expenditure, we can assess the efficiency of the termination of CSRs.

## Data availability statement

The original contributions presented in the study are included in the article/Supplementary material, further inquiries can be directed to the corresponding author.

## Author contributions

FL: Conceptualization, Data curation, Formal analysis, Investigation, Methodology, Resources, Software, Validation, Visualization, Writing – original draft, Writing – review & editing.

## Footnotes

^1^The ACA Marketplace provides four kinds of insurance plans: platinum, gold, silver, and bronze. Generally, the platinum and gold plans have higher premiums, more comprehensive coverage ranges, and lower copayments than the silver and bronze plans.

^2^ https://www.cms.gov/data-research/statistics-trends-and-reports/marketplace-products/2015-marketplace-open-enrollment-period-public-use-files

^3^ https://www.census.gov/programs-surveys/saipe.html

^4^See Appendix for the premium increase distribution.

^5^The treated states include Alabama, Florida, Michigan, Nevada, New Hampshire, Ohio, Pennsylvania, South Carolina, Utah, Virginia, Wisconsin, Wyoming. The control states include states adopted policy type I, II and III. Specifically, type I includes South Dakota; type II includes Delaware, Indiana, Mississippi, West Virginia; and type III includes Alaska, Arizona, Arkansas, Georgia, Illinois, Iowa, Kansas, Louisiana, Maine, Missouri, Montana, Nebraska, New Jersey, New Mexico, North Carolina, Oregon, Tennessee, Texas,.

^6^Because Policy Type 1 does not allow for adjustments to insurance premium, it is significantly different than Policy Types 2 and 3. Therefore, in Appendix, I show the regression results after moving states with policy type 1 out of the control group.
