# Supplementary material for: The impact of terminating cost-sharing reductions payments on health insurance plan choices
Source: Front Public Health. 2024 May 10;12:1370563. doi: 10.3389/fpubh.2024.1370563 (PMC11116663; doi:10.3389/fpubh.2024.1370563)
Supplement: Supplementary file 1 [file Data_Sheet_1.docx]

**Appendix 1: Percentage premium increase in 2018.**


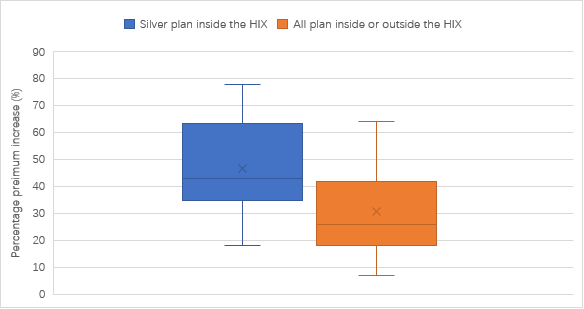


Source/Notes: Authors’ analysis of data for 2015–19 from the Open Enrollment Period Public Use Files. HIX represents the ACA health insurance exchange. The blue box shows the premium increases of the silver plan in treated states. The orange box shows the premium increases of the silver plan in control states, including states that increased the silver premium in and out of the marketplace and states increased all plans in and out of the marketplace.

**Appendix 2: The impacts of different price regulation policies on number of enrollees in each plan (excluding policy type I states).**

|  | (1) | (2) | (3) |
| --- | --- | --- | --- |
| Enrollment | Silver | Bronze | Gold&Platinum |
|  |  |  |  |
| Post*Treated | -355.20* | 465.81* | 12.71 |
|  | (189.32) | (255.26) | (65.91) |
| 200% FPL Income Ratio | 32,140.65*** | -40,932.80* | 14,716.83** |
|  | (10,532.43) | (23,620.86) | (6,086.74) |
| Population | -1,461.67 | 2,572.60 | -491.09 |
|  | (965.18) | (1,822.32) | (505.44) |
| Age | -34.27*** | -0.48 | -5.09 |
|  | (10.78) | (3.46) | (3.64) |
| White Prop | -253.92 | -661.68** | -50.83 |
|  | (237.06) | (259.10) | (87.44) |
|  |  |  |  |
| Observations | 12,224 | 12,224 | 12,224 |
| Mean | 2481.27 | 753.18 | 163.73 |

Source/Notes: Authors’ analysis of data for 2015–19 from the Open Enrollment Period Public Use Files. Table shows the did estimates of the impacts of different price regulation policies on number of enrollees in each plan from 2015 to 2019. Standard errors are reported in parentheses and clustered at the state level. *** p<0.01, ** p<0.05, * p<0.1.

**Appendix 3: Seemingly unrelated regressions**

As described in the empirical methods section, error terms might be correlated because once people choose one plan, the person cannot choose the other plans. Thus, I use the seemingly unrelated regression, which is a generalization of a linear regression model that allows errors to be correlated to check the robustness of my empirical methods. The estimates and significance levels are similar to those of the main regression, demonstrating the robustness of the empirical approach.

Table 5. Seemingly unrelated regressions for different price regulation policies.

|  | (1) | (2) | (3) |
| --- | --- | --- | --- |
| Enrollment | Silver | Bronze | Gold&Platinum |
|  |  |  |  |
| Post*Treated | -384.41* | 437.08* | 4.86 |
|  | (225.39) | (239.80) | (67.97) |
| 200% FPL Income Ratio | 371.13*** | -165.82*** | 50.72*** |
|  | (113.36) | (45.12) | (12.12) |
| Population | -3262.46*** | 1183.35*** | 287.24*** |
|  | (699.97) | (212.11) | (40.21) |
| Age | -13.91*** | -47.02** | -14.71*** |
|  | (49.87) | (16.90) | (4.34) |
| White Prop | -77.44*** | -25.49*** | -44.54*** |
|  | (30.81) | (79.2) | (123.10) |
|  |  |  |  |
| Observations | 12,489 | 12,489 | 12,489 |
| R-squared | 0.99 | 0.95 | 0.81 |

Note: Following Weesie (2000), I first do equation 1. Then, use postestimation command to report SUR results. Standard errors are reported in parentheses. Standard errors are reported in parentheses and clustered at the state level. *** p<0.01, ** p<0.05, * p<0.1.

Table 6. Seemingly unrelated regression results for the impacts of income proportions.

|  | 200% Ratio in Each County | 200% Ratio in Each Exchange |
| --- | --- | --- |
| Post·Proportion for the Silver plan | -2.30***  (0.11) | -0.38***  (0.01) |
| Post·Proportion for the Bronze plan | 1.54***  (0.12) | 0.32***  (0.01) |
| Post·Proportion for the gold and platinum plan | 0.21  (0.98) | 0.04  (0.11) |

Notes: Standard errors are reported in parentheses and clustered at the state level. *** p<0.01, ** p<0.05, * p<0.1.
